# Ocean acidification ameliorates harmful effects of warming in primary consumer

**DOI:** 10.1002/ece3.3526

**Published:** 2017-11-29

**Authors:** Sindre Andre Pedersen, Anja Elise Hanssen

**Affiliations:** ^1^ Department of Biology Faculty of Natural Sciences and Technology NTNU—Norwegian University of Science and Technology Trondheim Norway; ^2^ Library Section for Medicine and Health Sciences NTNU University Library NTNU—Norwegian University of Science and Technology Trondheim Norway

**Keywords:** *Calanus*, CO_2_, multiple stressors, ocean acidification, pH, reproduction, warming, zooplankton

## Abstract

Climate change‐induced warming and ocean acidification are considered two imminent threats to marine biodiversity and current ecosystem structures. Here, we have for the first time examined an animal's response to a complete life cycle of exposure to co‐occurring warming (+3°C) and ocean acidification (+1,600 μatm CO_2_), using the key subarctic planktonic copepod, *Calanus finmarchicus*, as a model species. The animals were generally negatively affected by warming, which significantly reduced the females’ energy status and reproductive parameters (respectively, 95% and 69%–87% vs. control). Unexpectedly, simultaneous acidification partially offset the negative effect of warming in an antagonistic manner, significantly improving reproductive parameters and hatching success (233%–340% improvement vs. single warming exposure). The results provide proof of concept that ocean acidification may partially offset negative effects caused by warming in some species. Possible explanations and ecological implications for the observed antagonistic effect are discussed.

## 1 INTRODUCTION

Rapid warming and ocean acidification are considered two of the most important threats to marine biodiversity in the coming centuries (Maclean & Wilson, 2011). Although the CO_2_‐concentration (*f*CO_2_) and temperature are expected to rise together in the future ocean, most effect studies on climate change have only focused on the isolated effects of changes in these stressors (Kroeker et al., 2013). However, this approach ignores the possibility that simultaneous rise in temperature and *f*CO_2_ may trigger interactive effects. Multiple stressors can give rise to simple additive effects (i.e., the response to combined stressors equals the sum of the response to the stressors in isolation, but no significant interaction) or significant interactions in the form of synergistic effects (stress greater than the sum of the individual stressor effects) or antagonistic effects (decreased stress)(Folt, Chen, Moore, & Burnaford, 1999). Reviews on the combined effect studies of warming and acidification have revealed prevalence of interactive effects on processes as diverse as calcification, growth, photosynthesis, reproduction, and survival (Byrne & Przeslawski, 2013; Harvey, Gwynn‐Jones, & Moore, 2013; Przeslawski, Byrne, & Mellin, 2015). However, these reviews also revealed that most combined effect studies have adopted acute‐ or short‐term exposure scenarios focused on restricted parts of the examined species life history. The short time frames provide limited opportunity for compensatory processes, such as physiological acclimation, genetic adaptation, and carry‐over effects (Dupont, Dorey, Stumpp, Melzner, & Thorndyke, 2013; Thor & Dupont, 2015), and thus, it involves a high implicit risk of either over‐ or underestimating the overall sensitivity of the examined organisms. Despite voiced concerns regarding the low degree of ecological relevance of effect studies on climate change (Dupont & Portner, 2013), it still remains to examine how animals will respond to co‐occurring warming and acidification over a complete life cycle.

Zooplankton contribute importantly to the biological cycling of carbon and other elements in the ocean (Roemmich & McGowan, 1995). The subarctic planktonic copepod, *Calanus finmarchicus*, seasonally dominates the zooplankton biomass in the surface waters of the northern North Sea and the North Atlantic (Planque & Batten, 2000), where it plays a key role in linking energy transfer from primary production (i.e., phytoplankton) to higher trophic level predators, including commercially important fish species (Beaugrand, Brander, Lindley, Souissi, & Reid, 2003). A distinct feature of the *Calanus* species is their seasonal production of a central body sac filled with energy‐rich lipids (Lee, Hagen, & Kattner, 2006), which is utilized as energy during winter diapause, and to fuel egg production and spawning in anticipation of the spring algae blooms (Mayor, Anderson, Pond, & Irigoien, 2009).

The aim of this study was to investigate the cumulative response of *C. finmarchicus* following a complete life cycle of co‐exposure to warming (14°C/+3°C) and acidification conditions relevant for year 2,300 (+1,600 μatm CO_2_)(Caldeira & Wickett, 2003), while maintaining the animals under the realistic ecological context of restricted food supply. Based on a reported temperature optimum close to 11° for *C. finmarchicus* (Moller, Maar, Jonasdottir, Nielsen, & Tonnesson, 2012), and previous observations of negative responses to ocean acidification conditions relevant for year 2300 (Pedersen et al., 2014), we hypothesized that animals would suffer additional reduction in the performance of fitness‐related traits in response to long‐term exposure to co‐occurring warming and acidification.

To examine this possibility, cohorts of newly spawned eggs from a laboratory culture of *C. finmarchicus* were followed under controlled laboratory conditions until the animals had developed into the adult stage (rearing experiment), and further, during their reproduction (egg production and hatching experiment). The animals were exposed using a fully crossed replicated experimental design (*n* = 3 per treatment); control (400 μatm CO_2_ & 11°C), ocean acidification (+1,600 μatm CO_2_), warming (+3°C), and co‐occurring exposure to warming and ocean acidification (+1,600 μatm & +3°C). The concentration of algal feed was selected to mimic a field situation where food supply is often suboptimal (Hirst & Lampitt, 1998), and where these animals often struggle to cover their daily demand of algae (Mayor et al., 2009). The regime is also relevant to future scenarios where warming‐induced stratification may reduce nutrient transport into the photic zone and lead to reduced primary production (Behrenfeld et al., 2006).

## 2 MATERIALS AND METHODS

### 2.1 Rearing experiment

**Figure 1:**
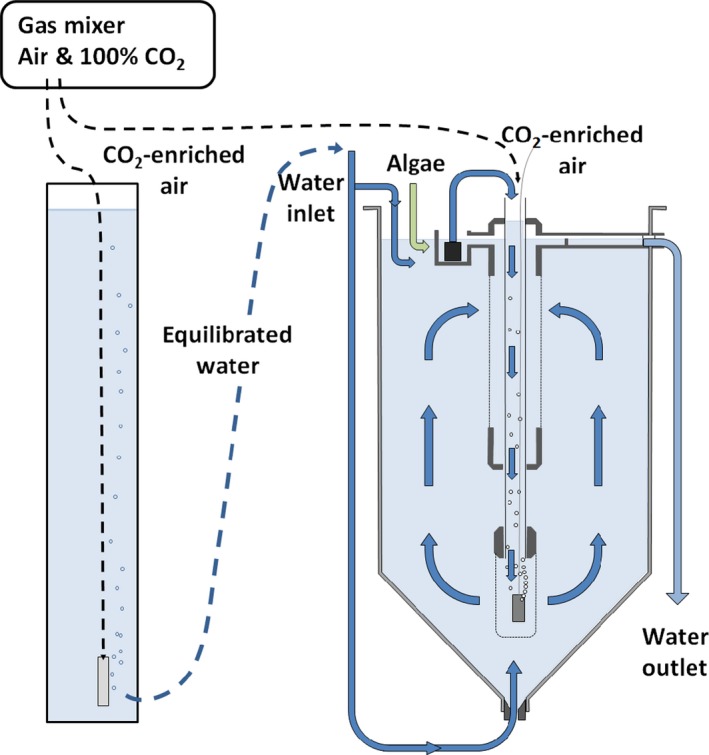
Schematic representation of the exposure system used in the rearing experiment. Water equilibrated by bubbling with normal air or CO_2_‐enriched air (produced by a gas‐mixer) was gravity feed to the top and base of the 90‐L exposure tanks, providing circulation. A secondary equilibration column was also integrated within each tank. A submersible pump, at the top of the secondary equilibration column, moved the water to improve the gas equilibration. A nylon mesh (120 μm, dashed lines) prevented the animals from entering the secondary equilibration system and from exiting the tanks

The animals were exposed using a fully crossed experimental design with three replicate tanks (90 L) for each treatment combination; control (400 μatm CO_2_ & 11°C), ocean acidification (+1,600 μatm CO_2_), warming (+3°C), and co‐occurring ocean acidification and warming (+1,600 μatm & +3°C). Newly spawned *C. finmarchicus* eggs were collected from an established culture maintained at 10°C and exposed under laboratory conditions, in a nonrecirculating flow‐through system with filtered (cutoff size 1 μm) natural seawater (see Figure 1 and Pedersen et al. (2014) for a closer description of the system). Each replicate tank was started with ~4,800 eggs which were subsequently continuously exposed to either of the four different treatment combinations for 65 days, until the animals had developed into the adult stage and reproduction commenced. To allow a gradual transition to the new target temperatures, the eggs were first incubated in 0.5‐L glass flasks that were submersed in the respective exposure tanks for circa two hours, before being released into the tanks. The hatched animals were continuously fed a mixture of unicellular algae; *Isochrysis galbana* (25 μg Carbon/L), *Dunaliella tertiolecta* (25 μg Carbon/L), and *Rhodomonas baltica* (200 μg Carbon/L). As *I. galbana* is considered too small to serve as prey for *C. finmarchicus* (Båmstedt, Nejstgaard, Solberg, & Høisœter, 1999), the total available carbon target concentration for was considered to be 225 μg Carbon/L, which is approximately 38% of the satiating carbon concentration reported for this species (Campbell, Wagner, Teegarden, Boudreau, & Durbin, 2001).

### 2.2 Egg production experiment

**Figure 2:**
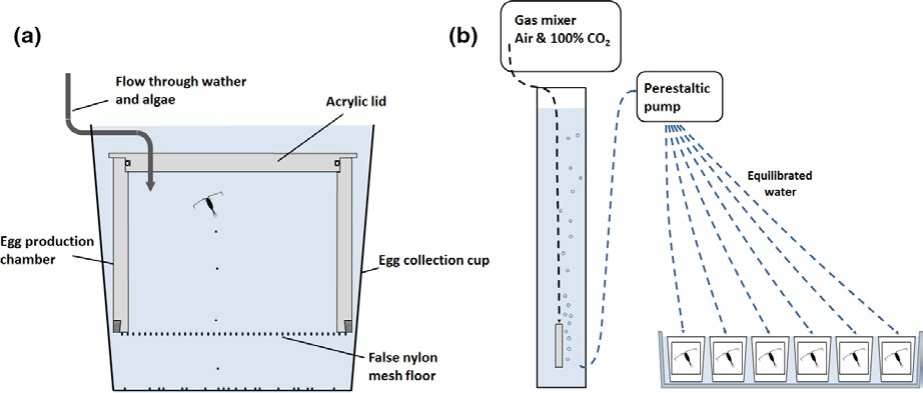
(a) Egg production chamber with airtight lid and a false nylon mesh floor (pore size: 300 μm) used to determine daily egg production rate of individual gravid females. Sinking, the spawned eggs collected on the floor of the egg collection cup. (b) Water equilibrated with the appropriate CO_2_‐concentration, temperature, and algae concentration was continuously distributed to the individual egg production chambers using multichannel peristaltic pumps. The egg production chambers were kept in temperature‐controlled water baths to maintain the correct temperature

When egg production commenced (at day 41 and 54 in the treatments exposed to 11 and 14°C, respectively), the fecundity in each replicate tank was estimated by measuring the daily egg clutch size (eggs female^−1^ day^−1^) in six gravid females over a five day period (the first day was ignored to avoid effects from handling stress). Females were transferred and maintained individually in egg production chambers (0.5 L) with a false floor that separated them from their sinking eggs, preventing cannibalism (see Figure 2a and Pedersen et al. (2014) for a closer description). The exposure condition (food, temperature, and carbon chemistry) was maintained similar to the respective treatment in the rearing experiment by means of a flow‐through system. The number of eggs produced, and their hatching success, was scored daily for each female by transferring the egg clutches to 8‐ml gas‐tight vials and incubating them under the same conditions as they were spawned. The incubation period was standardized according to the temperature conditions to allow 50% of the eggs sufficient time to hatch and develop into second larval stage (50 hr at 11°C and 26 hr 14°C), using a Belehrádek temperature equation developed for *C. finmarchicus* (Campbell et al., 2001). The eggs that remained unhatched after the incubation period were considered dead. Realized fecundity was found by multiplying the fecundity with the hatching success. These values were added together for each separate day to provide the cumulative realized fecundity of each female on a particular day of the egg production period. Following the last day of fecundity measurements, the females were sedated using MS 222 and photographed under a microscope to determine their body volume and lipid sac volume using standard morphometric techniques (Miller, Morgan, Prahl, & Sparrow, 1998). Due to lack of egg production, data on hatching success and lipid content could not be obtained on the females from one of the replicate tanks that received single warming exposure. The sex ratios in all replicate exposure tanks were also determined following the egg production experiment to identify potential fertilization issues due to a shortage of males.

### 2.3 Manipulation and verification of exposure conditions (*p*CO_2_, temperature, algae concentration)

In the rearing experiment, the CO_2_‐target concentrations were obtained by a combination of bubbling CO_2_‐enriched air into the exposure tanks and into the header tanks for the inlet water. A custom built gas‐mixing system with mass flow controllers was used to adjust the CO_2_‐concentration in the introduced air to obtain the target *p*CO_2_/pH in the water. The target temperatures in the treatments were maintained using aquarium heaters in the header tanks (200 W), and exposure tanks (50 W), regulated by PID‐controllers (SYL‐2372, Auber instruments, Alpharetta, Georgia, USA). Physical parameters were checked daily by measuring temperature (calibrated glass thermometer), salinity (saltwater refractometer), total scale pH (spectrophotometric determination according to Dickson, Sabine, and Christian (2007)), total alkalinity (open‐cell titration according to Xiaowan, Yunyan, Xiaoqiang, Chun, and Anping (2009)). Accuracy was verified by analyzing certified seawater (Scripps Institution of Oceanography, La Jolla, CA, USA). Carbonate system speciation was calculated using the software CO2SYS Version 2.1 (Lewis, Wallace, & Allison, 1998), with the dissociation constants for total scale of Mehrbach, Culberso, Hawley, and Pytkowic (1973), refitted by Dickson and Millero (1987).

In the egg production experiment, the CO_2_‐target concentrations were obtained in a similar manner as the rearing experiment, by bubbling CO_2_‐enriched air in header tanks used to supply the flow through water supplied to the egg production chambers (Figure 2b). The desired temperatures in the egg production chambers were obtained by immersing the egg production chambers in temperature‐controlled water baths and by controlling the temperature of the header tanks. Physical parameters were determined every other day by sampling the flow through water from the egg production chambers and measuring using the same methods as listed for the rearing experiment.

The algal concentration offered to the animals was measured on a daily basis using a coulter counter (MultisizerTM3 Coulter Counter^®^Beckman Coulter inc., USA). Filtered samples of the different algae (Whatman^®^ glass microfiber filters, Grade GF/C) were analyzed using a CHN‐analyzer (CHN Elemental Analyser 1106, Carlo Erba Instruments, Italy). Obtained carbon values were used to convert algal concentrations to carbon concentration equivalents.

The data were checked for normality using the Shapiro–Wilk test and transformed when necessary (values for hatching success were arcsine transformed, while the values for body volume, lipid sac volume, egg production, and realized fecundity were square‐root transformed). The biometric‐ and sex‐ratio data were analyzed using two‐way ANOVA. The egg production‐, hatching success‐, and realized fecundity data were analyzed using a generalized linear mixed‐effect model (GLMM), with maximum likelihood approximation. Temperature (two levels), *f*CO_2_ (two levels), and their two‐way interaction were treated as fixed effects. The algal measurements taken were averaged to define each tank's food availability and implemented as a fixed effect variable in the model. The random effect structure was day, repeated within each tank replicate. Model simplification was performed manually in a backward stepwise manner using AIC‐ and BIC values. Due to presence of significant interaction between temperature and *f*CO_2_, pairwise contrasts for the different treatment combinations were performed using the LSD test, after testing the groups for equal variance using Levene's test. The level of significance in all tests was set to 0.05, and all statistical analyses were performed using the statistical software SPSS (IMB^®^ SPSS^®^ Statistics, Version 21).

## 3 RESULTS

Both temperature and *f*CO_2_ remained relatively stable, and close to the target values, throughout the 62‐day‐long rearing experiment (Table 1). The algae concentration showed some variation between the individual exposure tanks, but a comparison revealed overlapping 95% confidence intervals (Table S1).

**Table 1:** Carbonate system speciation and temperature in the experimental treatments during the rearing experiment. The four treatments included control (400 μatm CO_2_/11°C), warming (+3°C), ocean acidification (+1,600 μatm CO_2_), and co‐occurring warming and ocean acidification (+1,600 μatm CO_2_/+3°C). Values for fugacity of CO_2_ (*f*CO_2_), in situ pH_T_, and calcium carbonate saturation state for calcite (ΩCa) were calculated from pH total scale (pH_T,25°C_), temperature, total alkalinity (*A*_T_), and salinity. Listed values represent means ± *SD* over the course of the experiment for the treatments, while the values presented within brackets show the number of replicate measurements taken any given time for the replicate tanks (*n* = 3)

Treatment	pH_T,25°C_	Temperature (°C)	*A*_T_	Salinity (PSU)	*f*CO_2_ (μatm)	pH_T,in situ_	Ω_Ca_
Control	7.77 ± 0.01(3)	11.1 ± 0.04(3)	2234 ± 85(1)	33.4 ± 0.4(1)	474 ± 25	7.97 ± 0.02	2.71 ± 0.17
Warming	7.78 ± 0.02(3)	14.0 ± 0.04(3)			514 ± 31	7.94 ± 0.02	2.84 ± 0.19
Acidified	7.22 ± 0.01(3)	11.1 ± 0.05(3)			2,021 ± 237	7.39 ± 0.03	0.79 ± 0.05
Warming * acidified	7.25 ± 0.02(3)	14.0 ± 0.04(3)			2,065 ± 154	7.38 ± 0.03	0.87 ± 0.08

**Figure 3:**
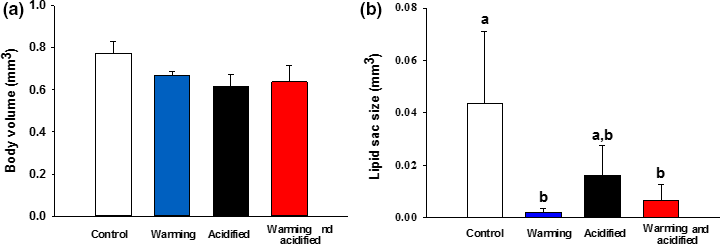
Body volume (mm^3^) (a) and central lipid sac volume (mm^3^) (b) of postspawning females: control (400 μatm CO_2_/11°C) (16 females), warming (+3°C) (4 females), ocean acidification (+1600 μatm CO_2_) (18 females), and co‐occurring warming and ocean acidification (+1600 μatm CO_2_ & +3°C) (17 females)). Bars indicate mean ± SD, with *n* = 3 in all treatments except for the warming only treatment (*n* = 2). Significant differences (*p* < .05) between groups are indicated by different letters

The mean body volume among the females raised under control conditions was 0.77 mm^2^ and showed no significant difference compared to the other treatments (*F*_3,8_ = 1,988, *p* = .204) (Figure 3a). The sex ratio also showed no significant difference in the treatments (male/female ratio (mean ± SD); control (0.30 ± 0.06), warming (0.28 ± 0.10), acidified (0.30 ± 0.12), warming and acidified (0.44 ± 0.04)(*F*_3,8_ = 2.221, *p* = .163)). The mean size of the central lipid sac was 0.04 mm^2^ in the postspawning females raised under control conditions (Figure 3b). Increased temperature caused a near depletion of the postspawning females’ central lipid sac volume, in both the single warming exposure (98% reduction vs. control, *p* = .008) and the co‐occurring warming and acidification exposure (86% reduction vs. control, *p* = .018).

Physical conditions also remained relatively stable throughout the egg production experiment, although the mean CO_2_‐level in elevated *f*CO_2_ treatments (acidified; 1621 μatm CO_2_, co‐occurring warming, and acidification; 1724 μatm CO_2_) was somewhat lower than the preset target level of 2,000 μatm CO_2_ (Table S2). The overall mean alga concentration in the exposure tanks was also 29% below the preset target value of 200 μg carbon per liter, but overlapping 95% confidence intervals between the different treatments suggest that there were no systematic differences in the food provisioning. The mean algal concentration in the exposure tanks also failed to qualify as a factor in the optimized models explaining egg production, hatching success, and realized fecundity, which included temperature and *f*CO_2_ as fixed effects, and their interaction term, with tank identity as a random factor.

**Figure 4:**
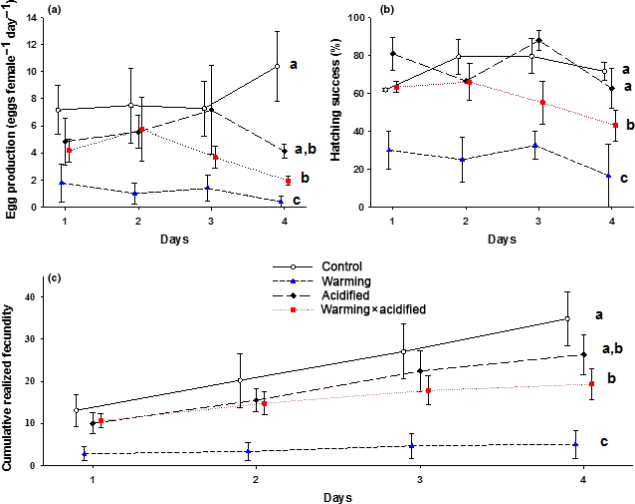
Fecundity (eggs female^−1^ day^−1^) (a), hatching success (%) (b), and cumulative realized fecundity (c) measured during a 4 days period in the different treatments; control (400 μatm CO_2_/11°C) (open circles), warming (+3°C) (blue triangles), ocean acidification (+1600 μatm CO_2_) (black diamonds), and co‐occurring warming and ocean acidification (+1600 μatm CO_2_ & +3°C) (read squares). Symbols and whiskers indicate tank mean (*n* = 3) and standard error. Different lower case letters indicate significant differences between the treatments in the shown period (*p* < .05)

The mean egg production among the females raised under control conditions was 7.54 (eggs female^−1^ day^−1^). Temperature and *f*CO_2_ had a significant interactive effect on the female egg production (*F*_1,12_ = 9.827, *p *<* *.01). Females raised under single warming conditions produced significantly fewer eggs compared to the females raised under the other treatments; 86% reduction vs. control, *p *<* *.001; 80% reduction vs. acidification, *p* = .009; 71% reduction vs. co‐occurring warming and acidification, *p* = .001 (Figure 4a). The egg production of females raised under co‐occurring warming and acidification was also significantly reduced compared to the control (52% reduction vs. control, *p* = .03).

The mean hatching success of the spawned eggs in the females raised under control conditions was 69%. A significant interaction between temperature and *f*CO_2_ was observed for the hatching success (*F*_1,11_ = 5.531, *p* = .038), with females raised under the single warming condition showing a marked reduction in hatching success compared to the other treatments; 69% reduction vs. control, *p *<* *.001; 70% reduction vs. acidification, *p* = .005; 57% reduction vs. co‐occurring warming and ocean acidification, *p *<* *.001 (Figure 4b). Eggs produced by females raised under co‐occurring warming and acidification also displayed a significantly reduced hatching success compared to the eggs of females developing under control‐ and acidified conditions (27% reduction vs. control, *p* = .028; 30% reduction vs. acidified, *p* = .016).

The mean realized fecundity in the females raised under control conditions was 7.0. Realized fecundity displayed a significant interaction between temperature and *f*CO_2_ (*F*_1,12_ = 10.067, *p* = .008), with females raised under single warming conditions showing a significant reduction compared to the other treatments; 87% reduction vs. control, *p *<* *.001; 83% reduction vs. acidified, *p *<* *.001, and 70% reduction vs. co‐occurring warming and acidification, *p* = .007 (Figure 4c). The females raised under co‐occurring warming and acidified conditions also had significantly lower realized fecundity than the control (57% reduction, *p* = .011).

## 4 DISCUSSION

The present study is unique in the sense that it is the first to assess the long‐term effects of co‐occurring warming and acidification in animals, by following a species over a whole life cycle (complete development from egg to the adult stage, and subsequent reproduction). The results indicate that cold‐water copepods will generally be much more affected by warming than ocean acidification. However, our study also provide proof of concept that the CO_2_‐concentration may interact with temperature in a manner that may partly offset the effect ocean warming: Acidification had no apparent effect on the examined life‐history traits when the animals were raised at 11°C, but significantly improved the reproductive performance when the temperature was raised to 14°C.

Studies examining growth and development of *C. finmarchicus* above 12°C are rare and there is still limited information regarding the performance of this species under the warmer conditions that are expected to occur within its current distribution range by the end of the century. The feeding efficiency of *C. finmarchicus* under different temperatures has been shown to follow a dome‐shaped response, with an optimum around 11°C (Moller et al., 2012). Also, as terminal body size is negatively related to increasing temperature (Campbell et al., 2001), and feeding efficiency is reduced by diminishing body size (Wirtz, 2012), higher temperatures are known to reduce the overall feeding efficiency of copepods. The reduction in lipid storage observed among the females raised at 14°C in the present study was therefore likely linked to a reduction in feeding efficiency. Moreover, long‐term exposure of *Calanus* has also shown that the metabolic rate of animals increases with increasing temperature (Hildebrandt, Niehoff, & Sartoris, 2014), resulting in a higher energy demand. The apparent failure to build energy reserves observed among the females raised at 14°C thus probably reflect the net result of both reduced energy intake and higher energy demands.

The egg production in *Calanus* is known to be influenced by factors such as temperature (Hirche, Meyer, & Niehoff, 1997), body size (Campbell & Head, 2000), and the quality and quantity of food (Campbell et al., 2001; Diel & Tande, 1992). *Calanus* species also rely heavily on their stored lipids as reserves to complete gonad maturation (Richardson, Jonasdottir, Hay, & Christoffersen, 1999), and poor nutritional female status can result in latent carry‐over effects such as reduction in both the size and hatching success of their eggs (Guisande & Harris, 1995). The reduced hatching success observed among the females raised at 14°C in the present study is therefore likely to reflect a maternal effect, where depleted female lipid reserves lead to production of fewer eggs and reduced offspring quality. A direct harmful effect of raising the temperature to 14°C on hatching success is more unlikely, as the hatching success of *C. finmarchicus* has been shown to be unaffected by incubation temperatures up to 19°C (Preziosi & Runge, 2014). As ingestion‐ and egg production rates are strongly correlated in calanoid copepods (Peterson & Dam, 1996), the dome‐shaped relationship between ingestion and temperature in *C. finmarchicus* (Moller et al., 2012) could also be expected to produce a similar relationship between temperature and egg production rate, as observed for a large number of other zooplankton species (Beaugrand, 2009; Halsband‐Lenk, Hirche, & Carlotti, 2002; Holste & Peck, 2006). The results in the present study are consistent with this notion, but a better resolved temperature relationship, based on more temperature points, is necessary before this relationship can be established for *Calanus* species.

The finding that long‐term acidification interacted with warming in a manner that ameliorated the negative effects of elevated temperature was contrary to our original hypothesis and also contrasts with the results from previous studies on the combined effects of warming and acidification in *Calanus* species. Five days acute exposure of a wild‐caught mixture of *C. finmarchicus* and *C. helgolandicus* subadults (C5 stage) to end of the century warming‐ (+2°C) and acidification scenarios (+700 μatm CO_2_) revealed no interactive effect between the two stressors on the examined life‐history traits (Mayor, Sommer, Cook, & Viant, 2015). Similar results were also observed in another study examining the combined effect of warming (0°C, +5°C, and +10°C) and acidification (390 μatm and +2600 μatm CO_2_) during a 30‐day exposure of wild‐caught females of *Calanus hyperboreus* (Hildebrandt et al., 2014). The absence of interactive effects in these studies may however reflect that moderate changes in the net energy accumulation efficiency may require longer exposure scenarios in order to manifest.

We previously observed a negative linear relationship between *f*CO_2_ and *C. finmarchicus* body size in in a multigenerational study conducted under restricted food availability at 10°C (Pedersen et al., 2014). However, a similar long‐term study on the same species conducted by another group under ad libitum food conditions failed to find negative effects of acidified conditions at 12°C, and instead reported an overall positive effects on body size across the different life stages (Runge et al., 2016). Interestingly, the modulating effect of CO_2_ on the temperature response observed in the present study may perhaps present an explanation for the conflicting results. When viewed together, the results from the present study, and the studies mentioned above, indicate that the response could be temperature dependent. At temperatures below 11°C, the response to acidification appears to be negative, while at higher temperature, the response seems to be positive.

Interestingly, the results in the present study are in line with findings in a recent mesocosm experiment, where nauplii production was observed to increase in response to simultaneous exposure to warming and acidification—a response that was attributed to the fertilizing effect of elevated *p*CO_2_ on the availability of phytoplankton prey (Garzke, Hansen, Ismar, & Sommer, 2016). Ocean acidification has been shown to affect copepod performance indirectly, by altering availably, community composition, or nutritional quality of their phytoplankton prey. Elevated *p*CO_2_ was found to reduce the content of polyunsaturated fatty acids in the diatom *Thalassiosira pseudonatna* and caused reduced egg production in *Acartia tonsa* (Rossoll et al., 2012). However, in a follow‐up study, no changes in the overall nutritional composition of the phytoplankton community from CO_2_‐induced acidification were observed by the same group (Rossoll, Sommer, & Winder, 2013). Measurement of the algal concentration throughout the present study does not suggest that the CO_2_‐concentration affected the abundance of the algae, and mean algal tank concentration was also found to be redundant as a factor in the models that best described the examined life‐history traits. A potential fertilizing effect of elevated *p*CO_2_ was attempted to be minimized in the experiment by the use of dim light conditions (restricting the potential for alga growth in the tanks) combined with a high turnover of the algae in the tank (resulting from a combination of high grazing intensity and flow through). However, despite these precautions, a CO_2_‐induced improvement of the algal nutritional quality cannot be excluded as a possible explanation for the positive effect of acidification that was observed at elevated temperature.

Experiments on acidification have revealed variable responses among taxa, species, populations, and experiments (see e.g., Kroeker et al. (2013) and Wittmann and Pörtner (2013)). Despite the apparent modulating effect of acidification, our results comply with the overall pattern that copepods appear to be relatively robust to near future acidification scenarios (but see also e.g., Fitzer et al. (2012)) and that these noncalcifying animals tend to be more sensitive toward rapid warming (Byrne & Przeslawski, 2013; Przeslawski et al., 2015). However, the context‐dependent response to acidification observed in this study opens up the possibility that acidification could negatively affect these animals at lower temperatures and should therefore be examined closer.

*Calanus finmarchicus* constitutes a key component of northern Atlantic food webs, with a large influence on the overall productivity of this ecosystem. However, over the last 50 years, the species distribution range has contracted, while the range of the more heat tolerant sibling species, *Calanus helgolandicus*, has expanded correspondingly. This shift has been linked to rising ocean temperatures (Hinder et al., 2014), and further retraction is expected to follow with the temperature increase expected to take place within year 2100 (Maar, Moller, Gurkan, Jonasdottir, & Nielsen, 2013). The hampering of reproductive performance observed among the animals raised under single warming conditions in the present study supports previous voiced concerns that warming will negatively affect the geographical range of cold‐water species (Harris, Edwards, & Olhede, 2014).

Simultaneously, modeling according to the RCP8.5 scenario indicates that surface waters may become up to two degrees warmer in the North Atlantic by year 2100, possibly increasing stratification and reducing the net primary productivity by up to 50% compared to historical values in some regions (Bopp et al., 2013), implying that copepods in these waters could struggle even more to cover their daily demand of algae. The authors also found that the pH could drop with up to 0.4 units in the North Atlantic by year 2100 (Bopp et al., 2013). However, the present study, conducted under conditions that resemble this situation (including restricted food conditions), reveals that acidification could perhaps ameliorate the negative effects of warming in an antagonistic manner that make the impact of warming less dramatic than previously expected for this species.

The results from our study thus provide proof of concept that acidification can significantly modulate the vital rates of our model species under future conditions, suggesting that it may be necessary to incorporate the effect of ocean acidification into current population dynamic models used to generate abundance and distribution scenarios for *C. finmarchicus*.

In summary, our results reveal that the performance of a cold‐water copepod species is reduced by end of the century projected warming, but we also find evidence suggesting that acidification could improve the reproductive performance of these animals under warmer conditions. This observation implies that the temperature‐driven northwards retraction of the investigated keystone species could be dampened by ocean acidification. The findings highlight the need to progress from short‐term and single‐stressor reductionist studies toward more long‐term studies that also incorporate multiple stressors, to improve the understanding the cumulative response of organisms to future climate change conditions, where multiple factors are expected to change gradually, and simultaneously, over the centuries to come.

## CONFLICT OF INTEREST

The authors declare that they have no competing interests.

## AUTHOR CONTRIBUTIONS

SAP designed the study, helped carry out the experiments, analyzed the data, and drafted the manuscript. AEH helped designing the study, carried out the experiments, analyzed the data, and assisted in writing the manuscript. All authors read and approved the final version of the manuscript.
