# Supplementary material for: Ocean acidification ameliorates harmful effects of warming in primary consumer
Source: Ecol Evol. 2017 Nov 29;8(1):396–404. doi: 10.1002/ece3.3526 (PMC5756865; doi:10.1002/ece3.3526)
Supplement: Supplementary file 1 [file ECE3-8-396-s001.docx]

Appendix 1

Table S1: Algal level (mean ± 95% confidence interval), expressed as carbon concentration (*μ*g Carbon/ L), prevailing during the rearing experiment and during the egg production experiment. The treatments are control (400 *μ*atm CO_2_/11˚C), warming (+3˚C ), ocean acidified (+1600 *μ*atm CO_2_) and co-occuring warming and ocean acidification (+1600 *μ*atm CO_2_/+3˚C).

|  |  | Algal concentration (*μ*g Carbon/ L) | | | |  | |
| --- | --- | --- | --- | --- | --- | --- | --- |
| Treatment |  | | Rearing experiment | Egg production experiment |  | |  |
| control |  | | 263.3±30.1 | 144.2±28.9 |  | |  |
| warming |  | | 227.7±29.9 | 170.6±34.9 |  | |  |
| acidified |  | | 246.1±35.8 | 153.1±62.8 |  | |  |
| warming * acidified |  | | 256.5±38.4 | 167.7±53.8 |  | |  |

Table S2: Carbonate system speciation and temperature in the experimental treatments during the 21 day long egg production experiment. Values represent means ±SD over the course of the experiment for the treatments and values listed within brackets indicate the number of replicate measurements taken at any given time. Values for fugacity of CO_2_ (*f*CO_2_), *in situ* pH_T_, and calcium carbonate saturation state for calcite (ΩCa) were calculated from pH total scale (pH_T,25˚C_), temperature and, total alkalinity (A_T_) and salinity. The treatments are control (400 *μ*atm CO_2_/11˚C), warming (+3˚C ), ocean acidified (+1600 *μ*atm CO_2_) and co-occuring warming and ocean acidification (+1600 *μ*atm CO_2_/+3˚C).

| Treatment | pH_T,25˚C_ | Temp | A_T_ | Salinity | *f*CO_2_ | pH_T,_*_in situ_* | Ω_Ca_ |
| --- | --- | --- | --- | --- | --- | --- | --- |
|  |  | (˚C) |  | (PSU) | (*μ*atm) |  |  |
| control | 7.79±0.01(2) | 11.1±0.01(2) | 2215±106(1) | 33.3±0.3(1) | 439±16 | 7.99±0.01 | 2.58±0.18 |
| warming | 7.80±0.01(2) | 14.0±0.01(2) |  |  | 474±58 | 7.96±0.02 | 2.70±0.19 |
| acidified | 7.30±0.03(2) | 11.1±0.03(2) |  |  | 1621±202 | 7.47±0.06 | 0.87±0.11 |
| warming*acidfied | 7.31±0.01(2) | 14.0±0.04(2) |  |  | 1724±156 | 7.45±0.02 | 0.82±0.05 |
